# 

**Published:** 1949-07

**Authors:** 


### CONTENTS

| | Page |
|---|---|
| **The Problem of Cut Flexor Tendons in Fingers.** By A. L. EYRE-BROOK. | 63 |
| **The Dramatic in Gynaecology and Obstetrics.** By V. B. GREEN-ARMYTAGE. | 69 |
| **Cholecystitis due to Liver Fluke.** By G. P. O'DONNELL. | 74 |
| **Some Diseases of the Newborn Baby.** By A. A. MONCRIEFF. | 77 |
| **Reviews of Books** | 80 |
| **Obituary** | 81 |
| **Meetings of Societies** | 82 |
| **Local Medical Notes** | 85 |

